# *Fagopyrum dibotrys* extract alleviates hepatic steatosis and insulin resistance, and alters autophagy and gut microbiota diversity in mouse models of high-fat diet-induced non-alcoholic fatty liver disease

**DOI:** 10.3389/fnut.2022.993501

**Published:** 2022-11-14

**Authors:** Dan Zhang, Yongfang Xu, Hang Chen, Da Wang, Zuotao Geng, Yuanli Chen, Yan Chen, Di Xiong, Rongna Yang, Xiaoting Liu, Yuke Zhang, Ping Xiang, Lanqing Ma, Jianjun Liu

**Affiliations:** ^1^Yunnan Key Laboratory of Stem Cell and Regenerative Medicine, Institute of Biomedical Engineering, Kunming Medical University, Kunming, China; ^2^The First Affiliated Hospital, Yunnan Institute of Digestive Disease, Yunnan Clinical Research Center for Digestive Diseases, Kunming Medical University, Kunming, China; ^3^The First People’s Hospital of Yunnan Province, Kunming, China; ^4^Lijiang Women and Children’s Hospital, Lijiang Maternity and Child Health Hospital, Lijiang, China; ^5^Faculty of Basic Medicine, Kunming Medical University, Kunming, China; ^6^School of Ecology and Environment, Institute of Environmental Remediation and Human Health, Southwest Forestry University, Kunming, China

**Keywords:** non-alcoholic fatty liver disease (NAFLD), *Fagopyrum dibotrys* extract, insulin resistance, autophagy, gut microbiota

## Abstract

Non-alcoholic fatty liver disease (NAFLD) is a major global health concern with increasing prevalence, with a lack of currently available effective treatment options; thus, the investigation of novel therapeutic approaches is necessary. The study aimed to investigate the outcomes and mechanisms of action of *Fagopyrum dibotrys* extract (FDE) in a high-fat diet (HFD)-induced mouse model of obesity. The findings showed that FDE supplementation attenuated glucose tolerance, insulin resistance (IR), hepatic steatosis, and abnormal lipid metabolism. In addition, FDE also promoted autophagic activity and inhibited the phosphorylation of transcription factor EB in HFD-fed mice. Furthermore, gut microbiota characterization via 16S rRNA sequencing revealed that the supplementation of FDE increased *Bacteroidetes* and *Verrucomicrobia* populations while decreased *Firmicutes*, thus modifying the gut microbiome. FDE also increased the relative abundance of *Akkermansia*. Our findings suggest that FDE may protect against HFD-induced NAFLD by activating autophagy and alleviating dysbiosis in the gut microbiome. FDE may be beneficial as a nutraceutical treatment for NAFLD.

## Introduction

Non-alcoholic fatty liver disease (NAFLD) is one of the main causes of chronic liver damage, which affects 25–35% of the population (1). Numerous medical disorders are associated with NAFLD, including hyperlipidemia, obesity, type 2 diabetes (T2DM), and cardiovascular disease (2). NAFLD, which can lead to a variety of conditions including non-alcoholic fatty liver (NAFL) and non-alcoholic steatohepatitis (NASH), can cause cirrhosis and liver cancer. The first line of defense against NAFLD is lifestyle modification, but a majority of patients struggle to make the necessary changes (3). Clinically, commonly used drugs against NAFLD including antidiabetic drugs, lipid-lowering drugs, antioxidants, insulin sensitizers, and liver-protecting drugs (4). Despite advancements in therapeutic target identification and drug development, no agent has been approved for this condition (5). A safe and effective drug or dietary strategy for treating NAFLD is urgently needed.

*Fagopyrum dibotrys* is a perennial herb that belongs to the *Fagopyrum* genus of the Polygonaceae family. Its rhizomes are used in traditional Chinese medicine and are effective in eliminating heat, toxicity, pus, sputum, and wind-dampness (6). Due to its notable anti-inflammatory, anti-oxidant, anti-cancer, anti-bacterial, and anti-diabetic activities, it has drawn considerable research attention in recent years which will likely expand its market and prospects (7). However, there is a lack of studies on *Fagopyrum dibotrys* and NAFLD. In this study, *Fagopyrum dibotrys* extract (FDE) was obtained from *Fagopyrum dibotrys* and its effect on NAFLD was studied, aiming to provide laboratory evidence for the application of *Fagopyrum dibotrys* in NAFLD.

The complex pathogenic mechanisms behind NAFLD are still not fully understood. Previous studies have indicated that several factors such as lipotoxicity, endoplasmic reticulum stress, oxidative stress, and dysbiosis of the gut microbiota contribute to metabolic dysfunction, which induce NAFLD (8). Accumulating evidence suggests that hepatic autophagy influences the physiological processes of the liver (9). Hepatic lipid metabolism relies heavily on autophagy. Schisandrin B is one of the most active dibenzocyclooctadiene isolated from Schisandra chinensis (Turcz.), which reduced hepatic steatosis by activating autophagy via the AMPK/mTOR pathway (10). A 5-week treatment of empagliflozin (the sodium-glucose co-transporter 2 inhibitor) attenuates NAFLD development by promoting autophagy and inhibiting hepatic apoptosis (11). Autophagic dysfunction may also contribute to hepatic steatosis and accelerate NAFLD progression (12). It can be seen that autophagy activation plays a key role in improving NAFLD. Whether FDE affects autophagy in NAFLD is still unclear, which deserves further study.

Recent studies have suggested that NAFLD pathogenesis in humans is closely associated with intestinal dysbiosis. Intestinal dysbiosis can accelerate NAFLD onset and development due to abnormal levels of microbial metabolites and their effects on intestinal permeability (13, 14). Thus, the gut microbiome may contain potential biomarkers for early diagnosis of NAFLD (15). This study analyzed the gut microbiota to determine the effects of FDE on its composition in mouse models of NAFLD.

In this study, we aimed to investigate the effects of FDE supplementation on hepatic steatosis and insulin resistance in mice with NAFLD. To further study the effects of FDE supplementation on autophagic activity of liver tissue and diversity of the gut microbiota in NAFLD mice. This will offer a theoretical foundation for the practical use of FDE as a dietary or therapeutic intervention for NAFLD.

## Materials and methods

### *Fagopyrum dibotrys* extract preparation

Fresh plant materials collected from the GAP planting base of *F. dibotrys* (Qujing, Yunnan Province, China) were analyzed by the Kunming Institute of Botany, Chinese Academy of Sciences. A fresh *F. dibotrys* plant (1000 g) was washed, cut up, and dried in the shade, and yielded approximately 200 g of powder. Hundred gram of dried powder was milled and extracted with 60% EtOH at 90°C (three extractions, 1 h per extraction) to obtain the crude extract. The crude extract was then eluted with 60% ethanol and filtered using polyamide column chromatography. The eluted ethanol extract was concentrated by rotary evaporation at 40°C, resulting in an extract of *F. dibotrys* (36 g) as a standard (16).

### Analysis of the nutrient composition of the *Fagopyrum dibotrys* extract

Metabolomics analysis was performed by Applied Protein Technology Co. Ltd. An appropriate sample was added to precooled methanol/acetonitrile/aqueous solution (2:2:1, v/v) and vortex-mixed. Following ultrasound for 30 min, the mixed sample was kept at –20°C for 10 min, and then centrifuged (14,000 *g*, 4°C) for 20 min. The supernatant was vacuum-dried and redissolved in 100 mL acetonitrile solution (acetonitrile: water = 1:1, v/v), and then centrifuged again at 14,000 *g* for 15 min at 4°C. In the end, the supernatant was analyzed by mass spectrometry (MS).

The samples were separated by an ultra-high performance liquid chromatography (UHPLC) system (Agilent, Santa Clara, CA, United States). The column temperature is set to 25°C; flow rate is 0.5 mL/min; injection volume is 2 μL. The mobile phase comprised of eluent A (water with 25 mM ammonium acetate and 25 mM ammonia) and eluent B (acetonitrile). The gradient elution method is used for chromatographic separation, and the gradient procedure is as follows: 0–0.5 min, 95% B; 0.5–7 min, from 95% B to 65% B; 7–8 min, from 65% B to 40% B; 8–9 min, 40% B; 9–9.1 min, from 40% B to 95% B; 9.1–12 min, 95% B.

MS/MS was conducted in both positive and negative ion modes using electrospray ionization (ESI) on AB Triple TOF 6600 (AB Sciex, California, CA, United States). The ESI source condition was set as follows: Ion Source Gas1 (60 psi), Ion Source Gas2 (60 psi), Curtain gas (30 psi), Ion source temperature: 600°C, spray voltage (ISVF) ± 5500 V (plus and minus two detection range of primary mass-to-charge ratio: 60–1000 Da, detection range of mass-to-charge ratio of secondary product ions: 25–1000 Da). Declustering voltage (DP): ± 60 V (both positive and negative modes), collision energy: 35 ± 15 eV. The information-dependent acquisition (IDA) conditions were: exclude isotopes within 4 Da, and candidate ions to monitor per cycle was 10.

### Animals

**FIGURE 1:**
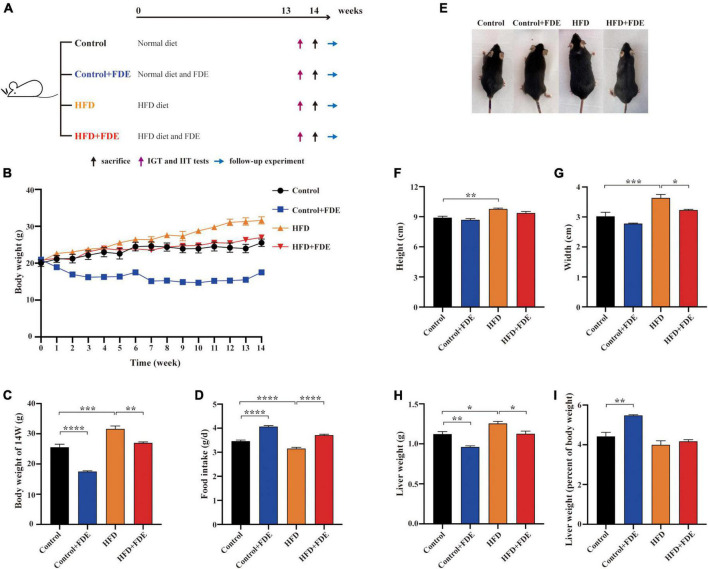
Effects of FDE on body weight and obesity in HFD-fed mice. **(A)** Experimental schedule. **(B)** Body weight change. **(C)** Body weight of male mice after feeding respective diets for 14 weeks. **(D)** Daily food intake (per day among the four groups). **(E)** Whole body image of male mice after feeding respective diets for 14 weeks. Effects of FDE on the body height **(F)** and width **(G)** of mice. **(H)** Liver weight and **(I)** liver index in each group. The data were analyzed by one-way ANOVA. All values are presented as the means ± SEM, and *n* = 4–6 per group. Control, normal diet; HFD, high-fat diet; Control + FDE, a mixture of FDE and the normal diet; HFD + FDE, a mixture of FDE and the high-fat diet. *P < 0.05, **P < 0.01, ***P < 0.001, ****P < 0.0001. HFD group versus the control group, HFD + FDE group versus the HFD group, or control + FDE group versus the control group.

Animal rearing and experimentation were performed in accordance with the China National Institutes of Health Guidelines for the Care and Use of Laboratory Animals. The Institutional Animal Care and Use Committee of Kunming Medical University approved all protocols (approval number kmmu20211584). Seven-week-old male C57BL/6 mice were purchased from Kunming Medical University. A normal diet (ND, 21033112; Keao Xieli Feed Co., Ltd, Beijing, China) and a high-fat diet (HFD, HD001b, 48.5% calories from fat; Becker Biotechnology Co., Ltd, Beijing, China) were used. Mice were housed in Kunming Medical University of Science and Technology’s Experimental Animal Center in controlled temperatures (22–25°C) and relative humidity (55–70%), under a 12 h light/dark cycle. The mice (18–22 g) were randomly divided into the following four groups (*n* = 6): control (mice received a normal diet); HFD (mice received a HFD); control + FDE group (mice received normal feed mixed with FDE at a mass ratio of 9:1); HFD + FDE group (mice received a HFD mixed with FDE at a mass ratio of 9:1). The composition of the diets is summarized in Supplementary Table 1. The high-fat diet and FDE were kept at 4°C for 1 month. After 1 week of adaptive feeding in mice, the different dietary interventions described above were started. All the mice were sacrificed after 14 weeks of different dietary interventions (Figure 1A).

### Food intake

Food intake was estimated using the following formula:

Food intake (g/d) = [initial weight of food provided in each group (g/d) – final weight of recovered food in each group (g/d)]/number of mice in each group. (Three mice in one cage and two cages in total for each group).

### Blood biochemical analyses

Serum aspartate aminotransferase (AST), alanine aminotransferase (ALT), alkaline phosphatase (ALP), total cholesterol (TC), triglyceride (TG), low-density lipoprotein (LDL), and high-density lipoprotein (HDL) levels were determined using an autoanalyzer (Cobas 6000 c501; Roche Diagnostics, Basel, Switzerland).

### Histology

Liver tissues were cut into 5 mm sections after being embedded in OCT solution and being frozen in liquid nitrogen. For the Oil Red O experiment, sections were first stained at 60°C for 10 min using Oil Red O staining solution, rinsed with 60% isopropyl alcohol, and then re-stained with hematoxylin. Standard hematoxylin and eosin (H&E) staining protocols were followed when staining liver and epididymal adipose tissues with H&E. The Oil Red O Staining and H&E Staining Kit were purchased from Solarbio Science & Technology (G1120, G1261; Beijing, China). Images were analyzed using ImageJ software after acquisition with a Leica Aperio CS2 system.

### Quantitative real-time PCR

TRIzol reagent (10296010; Invitrogen, Camarillo, CA, United States) was used to extract total RNA, followed by reverse transcription using a First Strand Rapid Synthesis Kit (G3330; Servicebio, Wuhan, China) to generate cDNA. A LightCycler 480 Real-Time PCR system (Roche Applied Science, Mannheim, Germany) was used for RT-PCR, and SYBR Premix Ex Taq (TaKaRa, Dalian, China) was used as the real-time polymerase chain reaction template. Supplementary Table 2 lists the primers used for quantitative real-time PCR (qRT-PCR). *GAPDH was* chosen as a normalization gene, and the 2^–ΔΔCT^ method was used to quantify the relative changes in gene expression.

### Insulin resistance measurements

After 14 weeks of FDE administration, intraperitoneal glucose tolerance (IGT) and intraperitoneal insulin tolerance (IIT) tests were performed on mice which were fasted for 16 and 2 h. Blood glucose levels were measured at the indicated times after intraperitoneal injection of glucose (1.8 g/kg body weight; Macklin Biochemical Co. Ltd, Shanghai, China) and human insulin (0.5 U/kg body weight; Wanbang Biochemical Medicine Group Co. Ltd., Jiangsu, China). Measurements were obtained using tail vein blood and Fast Take Glucose (Sinocare GA-3, Changsha, China) tests.

### Western blotting

Liver tissue was homogenized in RIPA buffer. Proteins were separated using 10 or 12.5% sodium dodecyl sulfate-polyacrylamide gel electrophoresis (SDS-PAGE) (PG112, PG112; Yase company, Shanghai, China), and transferred to polyvinylidene fluoride (PVDF) membranes before being blocked for 2.5 h with 5% skim milk-TBST. The following antibodies were incubated with the blots: anti-p-TFEB (1:2000; bs-22337R; Bioss, Beijing, China), anti-p62/SQSTM1 (1:20,000; EPR4844; Discovery Drive, Cambridge Biomedical Campus, Cambridge, United Kingdom), anti-LC3B (1:1,000; #83506; Cell Signaling Technology, Beverly, MA, United States), and anti-GAPDH (1:2,000; #97166; Cell Signaling Technology, Beverly, MA, United States). After a subsequent incubation with secondary antibodies, the blots were imaged using an imaging system (Amersham Imager 600) and ECL substrate reagents (#32109, Thermo Scientific Science, Waltham, MA, United States). Quantification was performed using ImageJ (NIH).

### Transmission electron microscopy

Fresh liver tissues (volume 1 mm^3^) were fixed with 2.5% glutaraldehyde solution for 4 h (4°C), and then rinsed with 0.1 mol/L phosphate buffer (PB; PH 7.4) three times for 15 min each. Tissues were then fixed with 1% osmium solution for 2 h (4°C) and rinsed twice (5 min each) with 0.1 mol/L PB (PH 7.4). After dehydration via an alcohol gradient, samples were permeated overnight with pure resin and then embedded in Epon 812 resin. The resin block was placed into an ultrathin slicer to generate 60–80 nm slices and then placed on 150 mesh copper film (Fang Hua). The copper mesh was dyed for 8 min in 2% uranium acetate-saturated alcohol solution, and the autophagosomes were visualized using an HT7700 transmission electron microscope (Hitachi High Technologies, Tokyo, Japan).

### 16S rRNA gene analysis

Stool samples from the HFD group, HFD + FDE group, and control group were collected. The bacterial 16S rRNA gene amplicons were sequenced on a MiSeq platform. Prior to controlling and filtering for sequence quality, the PE reads generated by MiSeq sequencing were spliced in accordance with the overlap relationships. OTU cluster analysis and sample differentiation were performed before species taxonomy analysis was conducted. Based on OTU cluster analysis, indices such as Chao1 can be obtained to analyze the diversity of bacterial communities. The bacterial community structure at all levels can be obtained based on taxonomic species analysis. The results of the in-depth community structure and phylogenetic analyses were used for data visualization.

### Statistical analysis

#### 16S rRNA data analysis

Microbiota richness and alpha diversity were measured by the Chao1 index based on the genus profiles. According to the discrete assumption, box-plots were made using the maximum and minimum values of each group of microorganisms. A cylindrical accumulation map was made to reflect the richness of the flora at the phylum and genus levels in each group. Principal coordinates analysis (PCoA) based on Bray–Curtis distances was used to visualize beta diversity (between-habitat diversity) to determine differences in community composition and microbiotal abundance. Additionally, linear discriminant analysis of effect size (LEfSe) (17) was performed to calculate taxon abundance and to determine the differences among groups (linear discriminant analysis score > 2 and *P* < 0.05 were considered significant) (18).

### Other results

Data are shown as the mean ± standard error of the mean. Shapiro–Wilk and Brown–Forsythe tests were used to check for normality and equal variance in the data. Multiple groups were analyzed statistically using Student–Newman–Keuls tests with one-way analysis of variance (ANOVA). GraphPad Prism 9.0 software was used for statistical analysis. *P* < 0.05 was considered significant.

## Results

### Analysis of the nutrient composition of the *Fagopyrum dibotrys* extract

The ultra-high performance liquid chromatography-quadrupole time-of-flight mass spectrometry (UHPLC-Q-TOF-MS) technique was used. A total of 1185 metabolites were detected, including 698 metabolites in positive ion mode and 487 metabolites in negative ion mode. In order to show the classification of these metabolites more visually, we drew the corresponding pie chart as shown in Supplementary Figure 1. In the figure, different colors represent different metabolites, and the size of the area represents the proportion of metabolites. At the superclass level, mainly organic acids and derivatives, organoheterocyclic compounds, lipids and lipid-like molecules, benzenoids, phenylpropanoids and polyketides, organic oxygen compounds. At the class level, mainly carboxylic acids and derivatives, Benzene and substituted derivatives, Organooxygen compounds, Fatty acyls, and Flavonoids. At the subclass level, mainly amino acids, peptides, and analogs, carbohydrates and carbohydrate conjugates, flavonoid glycosides, benzoic acids and derivatives and fatty acids and conjugates. Among them, phenolic compounds accounted for 7.8 and 13.4% in positive ion mode and negative ion mode. Organic acids and derivatives accounted for 23.8 and 20.7% in positive and negative ion mode, respectively.

### *Fagopyrum dibotrys* extract supplementation alleviates obesity in high-fat diet-fed mice

To investigate the effects of FDE on NAFLD, male C57BL/6 mice were fed a HFD with or without FDE supplementation for 14 weeks (Figure 1A). At week 14, mice fed a HFD had higher body weights than mice fed a normal diet. However, FDE supplementation notably decreased the body weight of HFD-fed mice (Figures 1B,C). Consistent with changes in body weight, body height and width were significantly increased in HFD-fed mice compared with the control group (*P* < 0.01 and *P* < 0.001, respectively), but was attenuated in the HFD + FDE group (*P* < 0.05) (Figures 1E–G). Notably, food intake had a negative relationship with weight change (Figures 1B–D). In addition, compared to the HFD group, liver weight was greatly reduced with FDE treatment (*P* < 0.05), but the liver index (liver weight/body weight) was not significantly changed (Figures 1H,I). In summary, these findings suggest that FDE supplementation alleviates HFD-induced obesity in mice.

### *Fagopyrum dibotrys* extract supplementation improved hepatic steatosis and lipid metabolism in high-fat diet-fed mice

**FIGURE 2:**
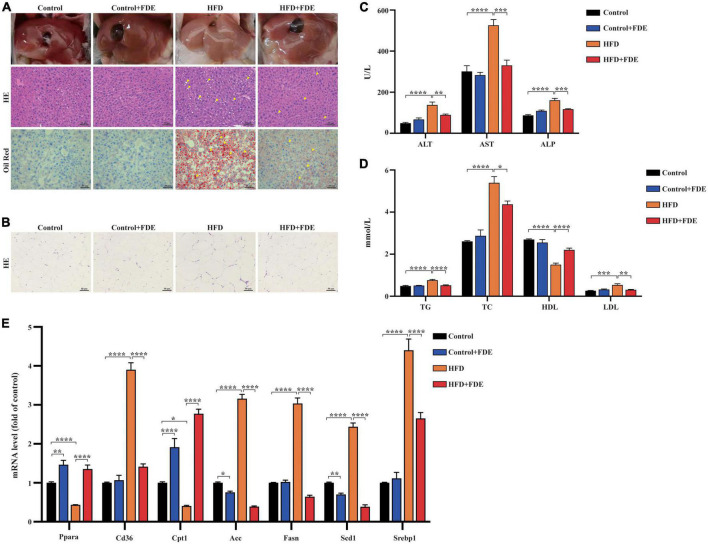
Effects of FDE on hepatic steatosis and lipid metabolism in HFD-fed mice. **(A)** Macroscopic image (top), H&E staining (middle), and Oil Red O staining (bottom) of liver sections of the four groups after feeding the respective diets for 14 weeks (magnification 200×; scale bar, 50 μm). The arrows indicate the fat vacuoles or lipid droplets. **(B)** Representative images of H&E staining of epididymal adipose tissue sections (magnification 200×; scale bar, 50 μm). Serum levels of **(C)** ALT, AST, ALP and **(D)** lipids in the four groups at the 14th week. **(E)** Quantitative real-time PCR analysis of the mRNA levels of lipid metabolism-related genes in the liver tissues. The data were analyzed by one-way ANOVA. All values are presented as the means ± SEM, and *n* = 3–6 per group. Control, normal diet; HFD, high-fat diet; Control + FDE, a mixture of FDE and normal diet; HFD + FDE, a mixture of FDE and high-fat diet. *P < 0.05, **P < 0.01, ***P < 0.001, ****P < 0.0001. HFD group versus the control group, HFD + FDE group versus the HFD group, or control + FDE group versus the control group.

Hepatic steatosis was evaluated via H&E and Oil Red O staining in HFD-induced obese mice to investigate the effects of FDE on hepatic steatosis and fat deposition. FDE supplementation significantly reduced hepatic steatosis and lipid accumulation (Figure 2A). The size of epididymal adipocytes was significantly larger in HFD-fed mice than in control mice, which was reduced by FDE supplementation in the HFD + FDE group (Figure 2B). Further validation of the protective effects of FDE was obtained by measuring the activity of liver marker enzymes. The data showed that HFD + FDE mice had lower serum ALT, AST, and ALP levels than HFD mice (Figure 2C). This suggests that FDE is potentially efficacious in improving abnormal liver function in HFD-fed mice. Furthermore, serum lipid levels, including TG, TC, and LDL, were decreased, while HDL levels were increased after FDE treatment (Figure 2D). Additionally, mRNA expression levels of lipid metabolism-related genes were also affected by FDE. The qPCR results showed that the expression of adipocyte genes involved in lipid uptake (*Cd36*) and lipogenesis (*Srebp1, Scd1, Fasn*, and *Acc*) were significantly increased in HFD-fed mice at 14 weeks. Administration with FDE to HFD-fed mice partially reversed these increases. Moreover, the expression of genes involved in fatty acid oxidation including *Ppar*α and *Cpt1* was decreased in HFD-fed mice, but FDE treatment significantly upregulated their expression (Figure 2E). These findings suggest that FDE may protect against HFD-induced steatosis and abnormal hepatic lipid metabolism.

### *Fagopyrum dibotrys* extract supplementation improved insulin resistance and glucose tolerance in high-fat diet-induced obese mice

**FIGURE 3:**
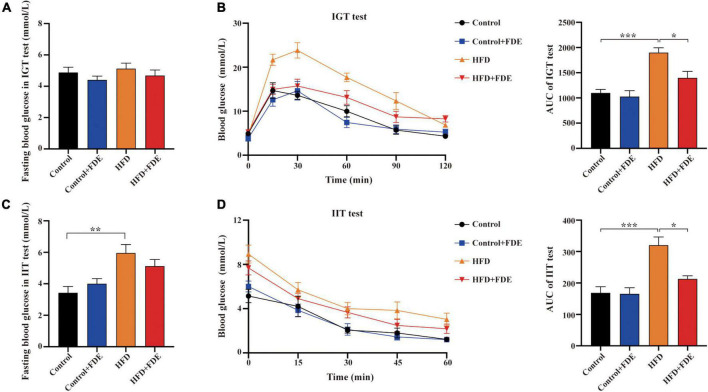
Effect of FDE on improving insulin resistance and glucose tolerance in HFD-fed mice. **(A)** Mean fasting blood glucose of each group after fasting for 16 h using the intraperitoneal glucose tolerance (IGT) test. **(B)** Left, IGT test performed on mice; right, the area under the curve (AUC) of IGT testing. **(C)** Mean fasting blood glucose of each group after fasting for 2 h in the intraperitoneal insulin tolerance (IIT) test. **(D)** Left, IIT test performed on mice; right, the AUC of IIT testing. The data were analyzed by one-way ANOVA. All values are presented as the means ± SEM, and *n* = 4–6 per group. Control, normal diet; HFD, high-fat diet; Control + FDE, a mixture of FDE and normal diet; HFD + FDE, a mixture of FDE and high-fat diet. *P < 0.05, **P < 0.01, ***P < 0.001. HFD group versus the control group, HFD + FDE group versus the HFD group.

High-fat diet-induced obese mice commonly develop obesity-related metabolic dysfunction such as insulin resistance and glucose intolerance. To explore whether FDE supplementation can improve this dysfunction, we conducted IGT and IIT tests. In comparison with the control group, HFD-fed mice had similar fasting blood glucose levels after a 16 h fast (*P* > 0.05) (Figure 3A). However, HFD-fed mice showed significantly elevated levels of fasting blood glucose after a 2 h fast (*P* < 0.01) (Figure 3C). In response to glucose and insulin, mice fed a HFD displayed higher blood glucose levels than mice fed a control diet. A significant reduction in blood glucose levels was observed in the HFD + FDE group compared to HFD alone (Figures 3B,D, left). The area under the curve (AUC) of the IGT and IIT tests was much higher in HFD-fed mice than in other groups (Figures 3B,D, right). Compared with the HFD group, the AUCs of the IGT and IIT tests were reduced by 26.4 and 33.5%, respectively, after FDE treatment (Figures 3B,D, right). Our findings suggest that FDE intervention improves insulin resistance in HFD-induced mouse models of NAFLD.

### *Fagopyrum dibotrys* extract supplementation promoted autophagic activity and reduced transcription factor EB phosphorylation in high-fat diet-fed mice

**FIGURE 4:**
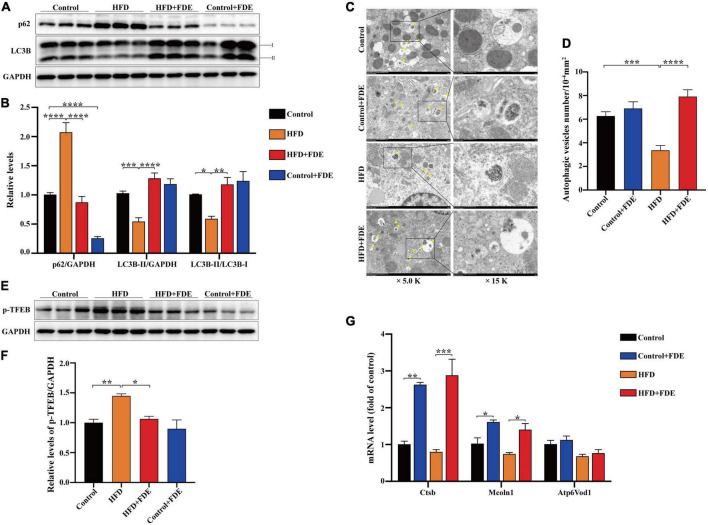
*Fagopyrum dibotrys* extract supplementation induces autophagy in the livers of mice with HFD-induced NAFLD. **(A,B)** Protein expression of P62 and LC3B, as detected by Western blot. **(C)** Representative transmission electron microscopy (TEM) images of autophagic vacuoles in hepatocytes (magnification × 5,000 or ×15,000; scale bar, 2.0 or 1.0 μm). The arrows indicate the autophagosome or autophagic vacuole. **(D)** Autophagic vesicles (arrow) were quantified in each group. **(E,F)** Protein expression of p-TFEB, as detected by Western blot. **(G)** Relative mRNA levels of TFEB target genes. The data were analyzed by one-way ANOVA. All values are presented as the means ± SEM, and *n* = 3–6 per group. Control, normal diet; HFD, high-fat diet; Control + FDE, a mixture of FDE and normal diet; HFD + FDE, a mixture of FDE and high-fat diet. *P < 0.05, **P < 0.01, ***P < 0.001, ****P < 0.0001. HFD group versus the control group, HFD + FDE group versus the HFD group, or control + FDE group versus the control group.

During autophagosome formation, lipidated microtubule-associated protein 1 light chain-3B (LC3B) transforms from LC3B-I to LC3B-II, which integrates into the outer membrane of autophagosomes. As an autophagy cargo receptor, sequestosome 1 (p62/SQSTM1) is recruited to the autophagosomal membrane via interaction with LC3B-II, and subsequently selectively targets ubiquitinated protein aggregates for autophagic degradation (19–22). Based on the above functions of LC3B and p62/SQSTM1, they have been widely used as markers for monitoring autophagic status. Recent studies have shown that autophagy regulates lipid metabolism; autophagy is inhibited in the livers of HFD-induced obese mice, and restoration of autophagy improves hepatic steatosis and increases insulin sensitivity (23). A study indicated that metformin alleviated lipid accumulation and IR by enhancing autophagic activity in HFD-fed mice (24). Thus, we hypothesized that FDE plays a protective role against NAFLD by inducing autophagy. To determine whether FDE supplementation induces autophagy in HFD-fed mice, we first measured the protein levels of LC3B and p62/SQSTM1 via Western blotting, and the results showed that FDE treatment significantly increased the protein level of LC3B-II and the LC3B-II/LC3B-I ratio while decreasing that of p62/SQSTM1 when compared with HFD-fed mice (Figures 4A,B) (*P* < 0.05). We next used transmission electron microscopy to observe autophagic vesicles, which possess a double membrane (25). Compared with the HFD group, the number of autophagic vesicles was increased significantly in the HFD + FDE group (Figures 4C,D) (*P* < 0.0001). These data suggest that FDE supplementation activates autophagy in HFD-induced obese mice. Transcription factor EB (TFEB), a positive regulator of autophagy, can activate the transcription of many autophagy- or lysosomal biogenesis-related genes (26). TFEB’s activity depends on its phosphorylation status; phosphorylated TFEB is inactive and located in the cytoplasm, while non-phosphorylated TFEB translocates to the nucleus and exerts its transcriptional activity by promoting expression of genes in the autophagy/lysosomal pathways (27, 28). Previous studies have indicated that the activity of TFEB is inhibited in HFD-induced obese mice. Activation of TFEB effectively improves insulin resistance, glucose intolerance, and hepatic steatosis in obese mice (24, 29). To determine whether FDE supplementation regulates TFEB activation, we measured phosphorylated cytoplasmic TFEB levels in each group, and the data showed that FDE supplementation decreased the phosphorylation level of TFEB in HFD-fed mice (Figures 4E,F). We also measured the mRNA levels of TFEB target genes. FDE treatment significantly upregulated the mRNA levels of *Ctsb* and *Mcln1* (Figure 4G). Taken together, these results suggest that FDE supplementation enhanced the activity of autophagy at least partially via TFEB activation.

### *Fagopyrum dibotrys* extract supplementation restructures intestinal microbial diversity in high-fat diet-fed mice

**FIGURE 5:**
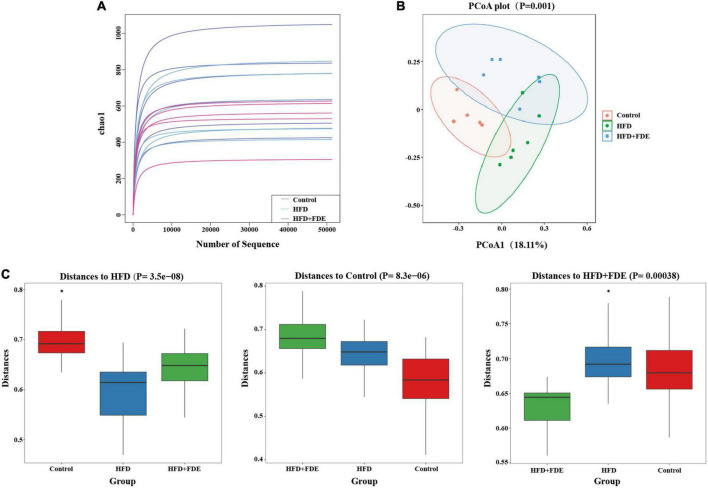
The composition of the intestinal flora of mice in each group. High-throughput sequencing of 16S rRNA was used to investigate the diversity of intestinal flora in the control, HFD, and HFD + FDE groups. OTU cluster analysis and taxonomic analysis were performed after identification. **(A)** Rarefaction curves show the species richness of each group using the Chao1 index. **(B)** The principal components analysis (PCoA) plot shows the similarity of microbial composition between samples. **(C)** Box plot showing the similarities or differences between groups. *n* = 5–6 per group. Control, normal diet; HFD, high-fat diet; Control + FDE, a mixture of FDE and normal diet; HFD + FDE, a mixture of FDE and high-fat diet.

**FIGURE 6:**
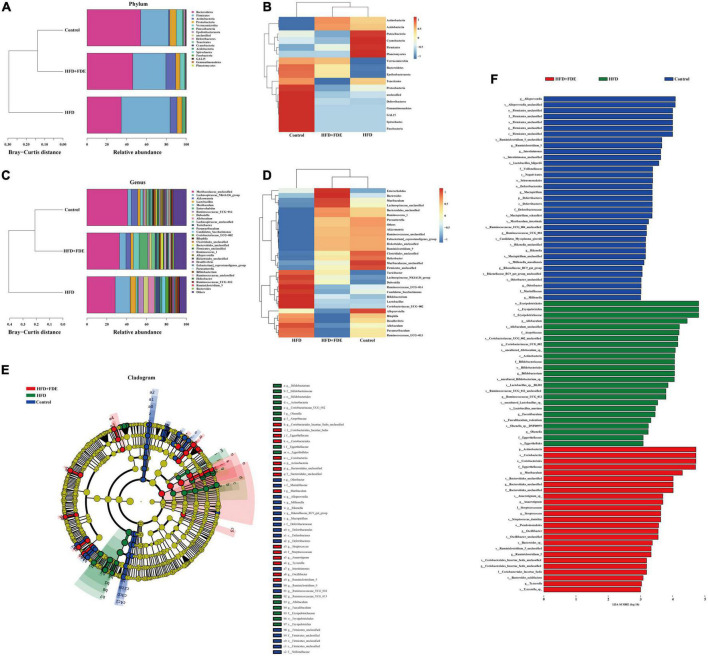
The relative abundance of intestinal flora in each group. **(A)** The relative abundance distribution map at the phylum level of each group. **(B)** Taxa heatmap at the phylum level. In the heatmap, the gradient from blue to red reflects the change from low to high abundance. **(C)** The relative abundance distribution map at the genus level of each group. **(D)** Taxa heatmap at the genus level. **(E)** Hierarchical tree diagram of LEfSe multilevel species, showing the taxonomy of the gut microbiota and its main bacterial structure. Circles radiating from the inside to the outside represent the phylum, class, order, family, genus. At different classification levels, each small circle represents a classification at that level. The diameter of the small circles is proportional to the relative abundance. Species with significantly different abundances between groups are colored in groups, while species with no significant differences are colored yellow. **(F)** Bar graph of LDA discriminant results, where the LDA value is >3.0 and the length of the bar graph represents the LDA value. *n* = 5–6 per group. Control, normal diet; HFD, high-fat diet; Control + FDE, a mixture of FDE and normal diet; HFD + FDE, a mixture of FDE and high-fat diet.

The gut microbiota plays an important role in the progression of NAFLD, and gut microbiota dysbiosis is a key factor in NAFLD pathogenesis (30–32). Based on 16S rRNA gene sequencing analysis, we investigated whether the gut microbiota was influenced by FDE treatment. We found that mice in the HFD group exhibited reduced gut microbial community richness (Chao1 index), which was alleviated after FDE intervention (Figure 5A). PCoA and boxplots of the microbiota showed clear differences between each group, while the FDE-treated group was similar to the control group (Figures 5B,C). We further explored the bacterial community composition of each group. There were four representative bacteria at the phylum level: *Firmicutes, Bacteroidetes, Verrucomicrobia*, and *Patescibacteria*. The relative abundance of *Bacteroidetes* and *Verrucomicrobia* was significantly elevated, while that of *Firmicutes* was reduced in the HFD + FDE group (Figures 6A,B). At the genus level, there was a significant difference between the HFD + FDE and HFD groups. The abundance of *Akkermansia, Muribaculaceae-unclassified*, and *Enterorhabdus* were dramatically increased, while *Lachnospiraceae-NK4A136-group* and *Lactobacillus* were decreased in the HFD + FDE group compared to the HFD group (Figures 6C,D).

A LEfSe analysis (logarithmic LDA score threshold = 3.0) was conducted for discriminative features in order to identify specific bacterial taxa after FDE supplementation. As high-dimensional biomarkers, 85 phylotypes from phylum to species were discovered among the three groups; specifically, the genera *Firmicutes, Deferribacteres*, and *Ruminiclostridium_9* were biomarkers in the control group. The genera *Bifidobacterium, Faecalibaculum*, and *Erysipelothrix* were predominant in the HFD group. *Bacteroidales, Oscillibacter, Ruminiclostridium_5*, and *Coriobacteriales* were biomarkers in the HFD + FDE group. Ultimately, our findings showed that FDE supplementation could significantly modulate the gut microbiota composition of HFD-fed mice (Figures 6E,F).

## Discussion

Non-alcoholic fatty liver disease etiology is intricate and complicated, and its underlying mechanisms remain unclear. Despite significant progress in understanding its epidemiology and etiology, there has been little progress in developing new therapeutics for this disease. NAFLD does not currently have a FDA-approved treatment, and the need for novel and effective therapeutic targets is high (33).

In this study, an experimental model of NAFLD was used to investigate the molecular mechanisms underlying FDE-induced amelioration of IR and hepatic steatosis. Based on our findings, FDE supplementation protects mice against HFD-induced NAFLD pathogenesis by activating autophagy. In addition, FDE supplementation affects the intestinal microbial diversity in HFD-fed mice by elevating the relative abundances of *Verrucomicrobia* and *Bacteroidetes*, while reducing that of *Firmicutes*. Therefore, this study provided clues about the underlying mechanisms of action of FDE when used as a NAFLD treatment.

In this study, we found that mice displayed a range of NAFLD-related metabolic and histological changes after 14 weeks of a HFD, including micro-and macro-vesicular steatosis, hepatic lipid accumulation, weight gain, impaired glucose tolerance, and increased insulin resistance. However, the administration of FDE to HFD-fed mice reduced body weight and liver weight, alleviated insulin resistance, and improved glucose tolerance. Moreover, FDE supplementation restored serum ALT, AST, and ALP levels. The lipid serum levels of TC, TG, and LDL were decreased, while HDL levels were increased by FDE supplementation. In our study, based on mRNA levels, FDE supplementation significantly upregulated the expression of *Ppar*α and *Cpt1* in hepatocytes, while significantly decreased the expression of *Cd36, Srebp1, Scd1, Fasn*, and *Acc*. These results suggest that FDE supplementation might inhibit fatty acid uptake and lipogenesis, and promote fatty acid oxidation, which may be beneficial in NAFLD. However, the specific underlying mechanisms need to be studied further. Additionally, FDE supplementation suppressed pathological changes in the livers of HFD-fed mice. The defensive efficacy of FDE supplementation against hepatic steatosis and insulin resistance was validated in this study.

*Fagopyrum dibotrys* Hara contains more than 100 identified compounds, primarily consisting of flavonoids, phenolics, terpenoids, steroids, and fatty acids (34). Flavonoids and phenolic compounds are regarded as the principal energetic elements of *F. dibotrys* Hara (34). There have been numerous *in vitro* studies and clinical trials that have demonstrated the beneficial effects of polyphenols on liver steatosis, as well as the associated pathogenic and clinical symptoms (35–37). Additionally, *in vitro* and *vivo* models of NAFLD have found that flavonoids and their derivatives have beneficial effects on the expression of genes related to lipid accumulation, oxidative stress, fibrosis, inflammation, and insulin resistance (38). To further understand the protective effect of FDE in NAFLD, we analyzed the nutrient composition of the FDE by UHPLC-Q-TOF MS and found that phenolic compounds accounted for 7.8 and 13.4% in positive ion mode and negative ion mode, respectively. The content of phenolic compounds was relatively low, and we were more concerned that other components in FDE might play a more important role in NAFLD. Interestingly, in the FDE we extracted, organic acids and derivatives accounted for 23.8 and 20.7% in positive and negative ion mode. Carboxylic acids, a huge class of organic acids, which account for a high proportion of FDE compounds (19.3% in positive mode; 15% in negative mode). The effects of FDE might be related to carboxylic acids. However, in the subclass analyses of FDE, we found that the presence of fatty acids is pretty low (4.3% in negative mode). In contrast to that the presence of amino acids is very higher (18.8% in positive mode) as such probably responsible for the high content of carboxylic acids and derivatives. Based on the composition complexity of FDE, the biological effects of FDE in NAFLD mice may not be related to the existence of one individual class or subclass of these compounds. It is the special pattern of the micro- and macrocomposition of the compound that plays a role in FDE treatment of NAFLD. However, the relevant mechanism of its improvement on NAFLD is still unclear, and further research is needed.

In autophagy, cytoplasmic self-degradation is carried out by releasing macromolecules and organelles into the lysosome, which is an evolutionary conserved degradation process. Autophagy is indispensable for maintaining biological homeostasis, and its dysfunction contributes to the pathogenesis of many diseases, as well as tissue injury, neurodegeneration, microbial infection, tumorigenesis, and aging (39). Autophagy is important for balancing energy sources and coping with nutritional stress at critical periods during development (40). It additionally performs the essential functions of removing misfolded or aggregated proteins, clearing damaged organelles, and removing intracellular pathogens (40). Evidence has suggested diminished hepatic autophagic flux during NAFLD development (41). According to several reports, autophagy inhibits NAFLD development by inducing fatty acid oxidation of stored intracellular lipids in hepatocytes (42, 43). Therefore, autophagy is crucial for preventing and improving NAFLD. Phloretin effectively attenuates the development of NAFLD by upregulating autophagy-mediated lipolysis, and the activation of autophagy is mainly manifested by decreasing the expression of SQSTM1/P62 and increasing the expression of Beclin1, Atg7, LC3 II/I, and Atg5 in liver tissues (44). In this study, we similarly found that FDE supplementation reduced the accumulation of SQSTMI/p62, an autophagic substrate, whose aggregation represents a reduction in autophagic flux (45). In addition, LC3B (LC3B-I and LC3B-II) regulates autophagy initiation. Mice with deficient autophagy show impaired conversion of LC3B-I to LC3B-II, resulting in a decreased level of autophagy (46). FDE supplementation reversed HFD-induced reductions of LC3B-II and the LC3B-II/LC3B-I ratio. These data suggest FDE-mediated alleviation of NAFLD may be via activation of the autophagic pathway.

A member of the microphthalmia family of simple helix-loop-helix leucine zipper transcription factors, TFEB regulates lysosomal function and autophagy (47). TFEB is a transcriptional regulator of the autophagy-lysosomal pathway that positively regulates gene expression related to autophagy and lysosomal biogenesis (48). In autophagic cells, activation of TFEB promotes the fusion of autophagosomes with lysosomes, the formation of autophagosomes, and the degradation of autophagic substrates (48). TFEB nuclear translocation is regulated via its phosphorylation (49). The mTOR complex 1 (mTORC1) phosphorylates TFEB on Ser211, causing the 14-3-3 proteins to bind to TFEB, which maintains the protein in its inactive cytosolic form (50). We found that FDE supplementation significantly reduces TFEB phosphorylation, promoting its activation.

Many elements can affect microbiota composition, including age, comorbid conditions, host genotype and exposure to antibiotics, and dietary habits (51). Specific dietary factors, such as macronutrient composition, diet type, or the presence of specific bioactive compounds have been shown to influence gut microbiota diversity and function (52). In addition, insoluble fiber, fat, and protein contents have important effects on the structure and function of the gut microbiota (53–55). Metabolic diseases such as NAFLD, diabetes, and obesity are often associated with the gut microbiota (56, 57). A dysregulated gut microbiome plays an important role in NAFLD pathogenesis via its metabolites. Therefore, restoring the gut microbiome and supplementing it with commensal bacteria may help reduce symptoms (58). The gut microbiota undergoes changes which have been linked to NAFLD progression. According to previous studies, the levels of *Bacteroidetes* decreased in NAFLD, while those of *Firmicutes* and *Proteobacteria* increased (59). A Mediterranean diet (MD), which is rich in polyphenols, polyunsaturated fats, vitamins, and carotenoids, promotes beneficial modifications of the gut microbiota, decreasing *Firmicutes* and increasing *Bacteroides* abundances. This has been proven to ameliorate obesity, infection, and associated metabolic alterations (60). Interestingly, in this study, at the phylum level, the relative abundances of *Bacteroidetes* and *Verrucomicrobia* were increased, while the levels of *Firmicutes* were reported to be decreased in the HFD + FDE group. A study showed that treatment with *Akkermansia muciniphila* could improve metabolic disorders caused by a HFD, including fat-mass gain, adipose tissue inflammation, metabolic endotoxemia, and insulin resistance (61). In this study, at the genus level, FDE supplementation dramatically increased the abundances of *Akkermansia, Muribaculaceae-unclassified*, and *Enterorhabdus*. Meanwhile, *Lachnospiraceae-NK4A136-group* and *Lactobacillus* were depleted. The therapeutic effect of FDE on NAFLD may be closely related to the changes in intestinal microbial diversity in HFD-fed mice. In addition, recent human and rodent research on obesity-related metabolic diseases have demonstrated the pivotal role of the gut microbiome in NAFLD pathogenesis (62, 63). FDE could be a potential nutraceutical for NAFLD by changing the composition of the gut microbiota. However, in this experiment, we were surprised to find that FDE could affect the daily food intake of mice. Food intake is a very important factor that affects the microbiome and many biological processes, such as the tricarboxylic acid (TCA) cycle and adenosine triphosphate (ATP) synthesis. These biological processes can also affect the diversity of gut microbiota (64, 65). So, the changes of gut microbiota after FDE supplementation will also be affected by these factors. Further studies are needed to clarify the direct effect of FDE on gut microbiota in NAFLD mice, which is still unclear.

## Conclusion

Our results demonstrated that FDE supplementation significantly alleviates hepatic steatosis, insulin resistance, and glucose tolerance in NAFLD. The efficacy of FDE for NAFLD may be due to autophagic activation and the restoration of the gut microbiota composition. Based on the findings of this study, we recommend that FDE can be considered as a potential nutraceutical for NAFLD; however, additional clinical research is needed to confirm our results.

## Data availability statement

The data presented in this study are deposited in the NCBI repository (www.ncbi.nlm.nih.gov), accession number PRJNA898514. Further inquiries can be directed to the corresponding authors.

## Ethics statement

The animal study was reviewed and approved by the Institutional Animal Care and Use Committee of Kunming Medical University (Approval no. kmmu20211584).

## Author contributions

DZ performed the data analysis and wrote the manuscript. YX, HC, DW, and ZG performed the investigation and collected the samples. YaC, DX, RY, XL, YZ, and PX performed the formal analysis, methodology, and software. YuC, JL, and LM performed the project administration and revised the manuscript. All authors contributed to the article and approved the submitted version.

## References

[1] Chhimwal J Patial V Padwad Y. Beverages and non-alcoholic fatty liver disease (NAFLD): think before you drink. *Clin Nutr.* (2021) 40:2508–19. 10.1016/j.clnu.2021.04.011 33932796

[2] Younossi ZM. Non-alcoholic fatty liver disease-A global public health perspective. *J Hepatol.* (2019) 70:531–44. 10.1016/j.jhep.2018.10.033 30414863

[3] Promrat K Kleiner DE Niemeier HM Jackvony E Kearns M Wands JR Randomized controlled trial testing the effects of weight loss on nonalcoholic steatohepatitis. *Hepatology.* (2010) 51:121–9. 10.1002/hep.23276 19827166 PMC2799538

[4] Huang DQ El-Serag HB Loomba R. Global epidemiology of NAFLD-related HCC: trends, predictions, risk factors and prevention. *Nat Rev Gastroenterol Hepatol.* (2021) 18:223–38. 10.1038/s41575-020-00381-6 33349658 PMC8016738

[5] Friedman SL Neuschwander-Tetri BA Rinella M Sanyal AJ. Mechanisms of NAFLD development and therapeutic strategies. *Nat Med.* (2018) 24:908–22. 10.1038/s41591-018-0104-9 29967350 PMC6553468

[6] Yang XW Zhang Y Ly LI. Advances in studies on medicinal plant of *Fagopyrum dibotrys*. *Mod Chin Med.* (2019) 21:837–46.

[7] Zhang LL He Y Sheng F Hu YF Song Y Li W Towards a better understanding of *Fagopyrum* dibotrys: a systematic review. *Chin Med.* (2021) 16:89. 10.1186/s13020-021-00498-z 34530893 PMC8447528

[8] Stefan N Häring H-U Cusi K. Non-alcoholic fatty liver disease: causes, diagnosis, cardiometabolic consequences, and treatment strategies. *Lancet Diabetes Endocrinol.* (2019) 7:313–24. 10.1016/S2213-8587(18)30154-2 30174213

[9] Ueno T Komatsu M. Autophagy in the liver: functions in health and disease. *Nat Rev Gastroenterol Hepatol.* (2017) 14:170–84. 10.1038/nrgastro.2016.185 28053338

[10] Yan LS Zhang SF Luo G Cheng BC Zhang C Wang YW Schisandrin B mitigates hepatic steatosis and promotes fatty acid oxidation by inducing autophagy through AMPK/mTOR signaling pathway. *Metabolism.* (2022) 131:155200. 10.1016/j.metabol.2022.155200 35405150

[11] Nasiri-Ansari N Nikolopoulou C Papoutsi K Kyrou I Mantzoros CS Kyriakopoulos G Empagliflozin attenuates non-alcoholic fatty liver disease (NAFLD) in high fat diet fed ApoE (-/-) mice by activating autophagy and reducing ER stress and apoptosis. *Int J Mol Sci.* (2021) 22:818. 10.3390/ijms22020818 33467546 PMC7829901

[12] Inami Y Yamashina S Izumi K Ueno T Tanida I Ikejima K Hepatic steatosis inhibits autophagic proteolysis via impairment of autophagosomal acidification and cathepsin expression. *Biochem Biophys Res Commun.* (2011) 412:618–25. 10.1016/j.bbrc.2011.08.012 21856284

[13] Song L Li Y Qu D Ouyang P Ding X Wu P The regulatory effects of phytosterol esters (PSEs) on gut flora and faecal metabolites in rats with NAFLD. *Food Funct.* (2020) 11:977–91. 10.1039/c9fo01570a 31803887

[14] Hu H Lin A Kong M Yao X Yin M Xia H Intestinal microbiome and NAFLD: molecular insights and therapeutic perspectives. *J Gastroenterol.* (2020) 55:142–58. 10.1007/s00535-019-01649-8 31845054 PMC6981320

[15] Tripathi A Debelius J Brenner DA Karin M Loomba R Schnabl B The gut-liver axis and the intersection with the microbiome. *Nat Rev Gastroenterol Hepatol.* (2018) 15:397–411. 10.1038/s41575-018-0011-z 29748586 PMC6319369

[16] Liang C Yuan JP Ding T Yan L Ling L Zhou XF Neuroprotective effect of fagopyrum dibotrys extract against Alzheimer’s disease. *Evid Based Complement Alternat Med.* (2017) 2017:3294586. 10.1155/2017/3294586 28512499 PMC5415668

[17] Segata N Izard J Waldron L Gevers D Miropolsky L Garrett WS Metagenomic biomarker discovery and explanation. *Genome Biol.* (2011) 12:R60. 10.1186/gb-2011-12-6-r60 21702898 PMC3218848

[18] Tian J Bai B Gao Z Yang Y Wu H Wang X Alleviation effects of GQD, a traditional Chinese medicine formula, on diabetes rats linked to modulation of the gut microbiome. *Front Cell Infect Microbiol.* (2021) 11:740236. 10.3389/fcimb.2021.740236 34692563 PMC8531589

[19] Cheong H Klionsky DJ. Chapter 1 biochemical methods to monitor autophagy-related processes in yeast. *Methods Enzymol.* (2008) 451:1–26. 10.1016/S0076-6879(08)03201-1 19185709

[20] Kabeya Y Mizushima N Ueno T Yamamoto A Kirisako T Noda T LC3, a mammalian homologue of yeast Apg8p, is localized in autophagosome membranes after processing. *EMBO J.* (2008) 19:5720–8. 10.1093/emboj/19.21.5720 11060023 PMC305793

[21] Schlafli AM Berezowska S Adams O Langer R Tschan MP. Reliable LC3 and p62 autophagy marker detection in formalin fixed paraffin embedded human tissue by immunohistochemistry. *Eur J Histochem.* (2015) 59:2481. 10.4081/ejh.2015.2481 26150155 PMC4503968

[22] Pankiv S Clausen TH Lamark T Brech A Bruun JA Outzen H p62/SQSTM1 binds directly to Atg8/LC3 to facilitate degradation of ubiquitinated protein aggregates by autophagy. *J Biol Chem.* (2007) 282:24131–45. 10.1074/jbc.M702824200 17580304

[23] Singh R Kaushik S Wang Y Xiang Y Novak I Komatsu M Autophagy regulates lipid metabolism. *Nature.* (2009) 458:1131–5. 10.1038/nature07976 19339967 PMC2676208

[24] Zhang D Ma Y Liu J Deng Y Zhou B Wen Y Metformin alleviates hepatic steatosis and insulin resistance in a mouse model of high-fat diet-induced nonalcoholic fatty liver disease by promoting transcription factor EB-dependent autophagy. *Front Pharmacol.* (2021) 12:689111. 10.3389/fphar.2021.689111 34366846 PMC8346235

[25] David CAW Del Castillo Busto ME Cuello-Nunez S Goenaga-Infante H Barrow M Fernig DG Assessment of changes in autophagic vesicles in human immune cell lines exposed to nano particles. *Cell Biosci.* (2021) 11:133. 10.1186/s13578-021-00648-8 34271993 PMC8283997

[26] Yin Z Pascual C Klionsky DJ. Autophagy: machinery and regulation. *Microb Cell.* (2016) 3:588–96. 10.15698/mic2016.12.546 28357331 PMC5348978

[27] Chen G Mu C Chen Y An N Zhu Y Kepp O Monitoring TFEB translocation. *Methods Cell Biol.* (2021) 164:1–9. 10.1016/bs.mcb.2020.10.017 34225908

[28] Sakellariou D Tiberti M Kleiber TH Blazquez L López AR Abildgaard MH eIF4A3 regulates the TFEB-mediated transcriptional response via GSK3B to control autophagy. *Cell Death Differ.* (2021) 28:3344–56. 10.1038/s41418-021-00822-y 34158631 PMC8630043

[29] Du X Di Malta C Fang Z Shen T Niu X Chen M Nuciferine protects against high-fat diet-induced hepatic steatosis and insulin resistance via activating TFEB-mediated autophagy-lysosomal pathway. *Acta Pharm Sin B.* (2022) 12:2869–86. 10.1016/j.apsb.2021.12.012 35755273 PMC9214335

[30] Li S Chen F Zou Y Ning L Zhang G Zhang S Yinzhihuang oral liquid protects against non-alcoholic steatohepatitis via modulation of the gut-liver axis in mice. *Ann Transl Med.* (2022) 10:631. 10.21037/atm-21-4809 35813333 PMC9263770

[31] Li P Hu J Zhao H Feng J Chai B. Multi-omics reveals inhibitory effect of baicalein on non-alcoholic fatty liver disease in mice. *Front Pharmacol.* (2022) 13:925349. 10.3389/fphar.2022.925349 35784718 PMC9240231

[32] Yao N Yang Y Li X Wang Y Guo R Wang X Effects of dietary nutrients on fatty liver disease associated with metabolic dysfunction (MAFLD): based on the intestinal-hepatic axis. *Front Nutr.* (2022) 9:906511. 10.3389/fnut.2022.906511 35782947 PMC9247350

[33] Raza S Rajak S Upadhyay A Tewari A Anthony Sinha R. Current treatment paradigms and emerging therapies for NAFLD/NASH. *Front Biosci.* (2021) 26:206–37. 10.2741/4892 33049668 PMC7116261

[34] Jing R Li HQ Hu CL Jiang YP Qin LP Zheng CJ. Phytochemical and pharmacological profiles of three fagopyrum buckwheats. *Int J Mol Sci.* (2016) 17:589. 10.3390/ijms17040589 27104519 PMC4849043

[35] Rodriguez-Ramiro I Vauzour D Minihane AM. Polyphenols and non-alcoholic fatty liver disease: impact and mechanisms. *Proc Nutr Soc.* (2016) 75:47–60. 10.1017/S0029665115004218 26592314

[36] Salomone F Godos J Zelber-Sagi S. Natural antioxidants for non-alcoholic fatty liver disease: molecular targets and clinical perspectives. *Liver Int.* (2016) 36:5–20. 10.1111/liv.12975 26436447

[37] Van De Wier B Koek GH Bast A Haenen GR. The potential of flavonoids in the treatment of non-alcoholic fatty liver disease. *Crit Rev Food Sci Nutr.* (2017) 57:834–55. 10.1080/10408398.2014.952399 25897647

[38] Pisonero-Vaquero S González-Gallego J Sánchez-Campos S García-Mediavilla MV. Flavonoids and related compounds in non-alcoholic fatty liver disease therapy. *Curr Med Chem.* (2015) 22:2991–3012. 10.2174/0929867322666150805094940 26242257

[39] Levine B Kroemer G. Biological functions of autophagy genes: a disease perspective. *Cell.* (2019) 176:11–42. 10.1016/j.cell.2018.09.048 30633901 PMC6347410

[40] Glick D Barth S Macleod KF. Autophagy: cellular and molecular mechanisms. *J Pathol.* (2010) 221:3–12. 10.1002/path.2697 20225336 PMC2990190

[41] Niture S Lin M Rios-Colon L Qi Q Moore JT Kumar D. Emerging roles of impaired autophagy in fatty liver disease and hepatocellular carcinoma. *Int J Hepatol.* (2021) 2021:6675762. 10.1155/2021/6675762 33976943 PMC8083829

[42] Samovski D Abumrad NA. Regulation of lipophagy in NAFLD by cellular metabolism and CD36. *J Lipid Res.* (2019) 60:755–7. 10.1194/jlr.C093674 30819696 PMC6446712

[43] Zhang Z Yao Z Chen Y Qian L Jiang S Zhou J Lipophagy and liver disease: new perspectives to better understanding and therapy. *Biomed Pharmacother.* (2018) 97:339–48. 10.1016/j.biopha.2017.07.168 29091883

[44] Chhimwal J Goel A Sukapaka M Patial V Padwad Y. Phloretin mitigates oxidative injury, inflammation and fibrogenic responses via restoration of autophagic flux in in-vitro and pre-clinical models of NAFLD. *J Nutr Biochem.* (2022) 107:109062. 10.1016/j.jnutbio.2022.109062 35609858

[45] Fukushima H Yamashina S Arakawa A Taniguchi G Aoyama T Uchiyama A Formation of p62-positive inclusion body is associated with macrophage polarization in non-alcoholic fatty liver disease. *Hepatol Res.* (2018) 48:757–67. 10.1111/hepr.13071 29473277

[46] Ma D Molusky MM Song J Hu CR Fang F Rui C Autophagy deficiency by hepatic FIP200 deletion uncouples steatosis from liver injury in NAFLD. *Mol Endocrinol.* (2013) 27:1643–54. 10.1210/me.2013-1153 23960084 PMC4061382

[47] Napolitano G Ballabio A. TFEB at a glance. *J Cell Sci.* (2016) 129:2475–81. 10.1242/jcs.146365 27252382 PMC4958300

[48] Song TT Cai RS Hu R Xu YS Qi BN Xiong YA. The important role of TFEB in autophagy-lysosomal pathway and autophagy-related diseases: a systematic review. *Eur Rev Med Pharmacol Sci.* (2021) 25:1641–9. 10.26355/eurrev_202102_24875 33629334

[49] Yan S. Role of TFEB in autophagy and the pathogenesis of liver diseases. *Biomolecules.* (2022) 12:672. 10.3390/biom12050672 35625599 PMC9139110

[50] Xu Y Ren J He X Chen H Wei T Feng W. YWHA/14-3-3 proteins recognize phosphorylated TFEB by a noncanonical mode for controlling TFEB cytoplasmic localization. *Autophagy.* (2019) 15:1017–30. 10.1080/15548627 30653408 PMC6526839

[51] Gómez-Hurtado I Santacruz A Peiró G Zapater P Gutiérrez A Pérez-Mateo M Gut microbiota dysbiosis is associated with inflammation and bacterial translocation in mice with CCl4-induced fibrosis. *PLoS One.* (2011) 6:e23037. 10.1371/journal.pone.0023037 21829583 PMC3146520

[52] Nadal I Santacruz A Marcos A Warnberg J Garagorri JM Moreno LA Shifts in clostridia, *Bacteroides* and immunoglobulin-coating fecal bacteria associated with weight loss in obese adolescents. *Int J Obes.* (2009) 33:758–67. 10.1038/ijo.2008.260 19050675

[53] Clemente JC Ursell LK Parfrey LW Knight R. The impact of the gut microbiota on human health: an integrative view. *Cell.* (2012) 148:1258–70. 10.1016/j.cell.2012.01.035 22424233 PMC5050011

[54] Muegge BD Kuczynski J Knights D Clemente JC González A Fontana L Diet drives convergence in gut microbiome functions across mammalian phylogeny and within humans. *Science.* (2011) 332:970–4. 10.1126/science.1198719 21596990 PMC3303602

[55] Richards JL Yap YA McLeod KH Mackay CR Mariño E. Dietary metabolites and the gut microbiota: an alternative approach to control inflammatory and autoimmune diseases. *Clin Transl Immunology.* (2016) 5:e82. 10.1038/cti.2016.29 27350881 PMC4910123

[56] Chen J Vitetta L. Bile acids and butyrate in the effects of probiotics/synbiotics on nonalcoholic fatty liver disease. *Eur J Gastroenterol Hepatol.* (2019) 31:1475–6. 10.1097/MEG.0000000000001506 31464781

[57] Chen J Vitetta L. Mitochondria could be a potential key mediator linking the intestinal microbiota to depression. *J Cell Biochem.* (2020) 121:17–24. 10.1002/jcb.29311 31385365

[58] Chen J Vitetta L. Gut microbiota metabolites in NAFLD pathogenesis and therapeutic implications. *Int J Mol Sci.* (2020) 21:5214. 10.3390/ijms21155214 32717871 PMC7432372

[59] Aron-Wisnewsky J Vigliotti C Witjes J Le P Holleboom AG Verheij J Gut microbiota and human NAFLD: disentangling microbial signatures from metabolic disorders. *Nat Rev Gastroenterol Hepatol.* (2020) 17:279–97. 10.1038/s41575-020-0269-9 32152478

[60] Anania C Perla FM Olivero F Pacifico L Chiesa C. Mediterranean diet and nonalcoholic fatty liver disease. *World J Gastroenterol.* (2018) 24:2083–94. 10.3748/wjgv24.i19.2083 29785077 PMC5960814

[61] Everard A Belzer C Geurts L Ouwerkerk JP Druart C Bindels LB Cross-talk between *Akkermansia* muciniphila and intestinal epithelium controls diet-induced obesity. *Proc Natl Acad Sci U S A.* (2013) 110:9066–71. 10.1073/pnas.1219451110 23671105 PMC3670398

[62] Chiu CC Ching YH Li YP Liu JY Huang YT Huang YW Nonalcoholic fatty liver disease is exacerbated in high-fat diet-fed gnotobiotic mice by colonization with the gut microbiota from patients with nonalcoholic steatohepatitis. *Nutrients.* (2017) 9:1220. 10.3390/nu9111220 29113135 PMC5707692

[63] Wang B Jiang X Cao M Ge J Bao Q Tang L Altered fecal microbiota correlates with liver biochemistry in nonobese patients with non-alcoholic fatty liver disease. *Sci Rep.* (2016) 6:32002. 10.1038/srep32002 27550547 PMC4994089

[64] Zhou J Tang L Shen CL Wang JS. Green tea polyphenols boost gut-microbiota-dependent mitochondrial TCA and urea cycles in sprague-dawley rats. *J Nutr Biochem.* (2020) 81:108395. 10.1016/j.jnutbio.2020.108395 32388254

[65] Belizário JE Faintuch J Garay-Malpartida M. Gut microbiome dysbiosis and immunometabolism: new frontiers for treatment of metabolic diseases. *Mediators Inflamm.* (2018) 2018:2037838. 10.1155/2018/2037838 30622429 PMC6304917

