# Supplementary figures and images for: Fagopyrum dibotrys extract alleviates hepatic steatosis and insulin resistance, and alters autophagy and gut microbiota diversity in mouse models of high-fat diet-induced non-alcoholic fatty liver disease
Source: Front Nutr. 2022 Nov 14;9:993501. doi: 10.3389/fnut.2022.993501 (PMC9704541; doi:10.3389/fnut.2022.993501)

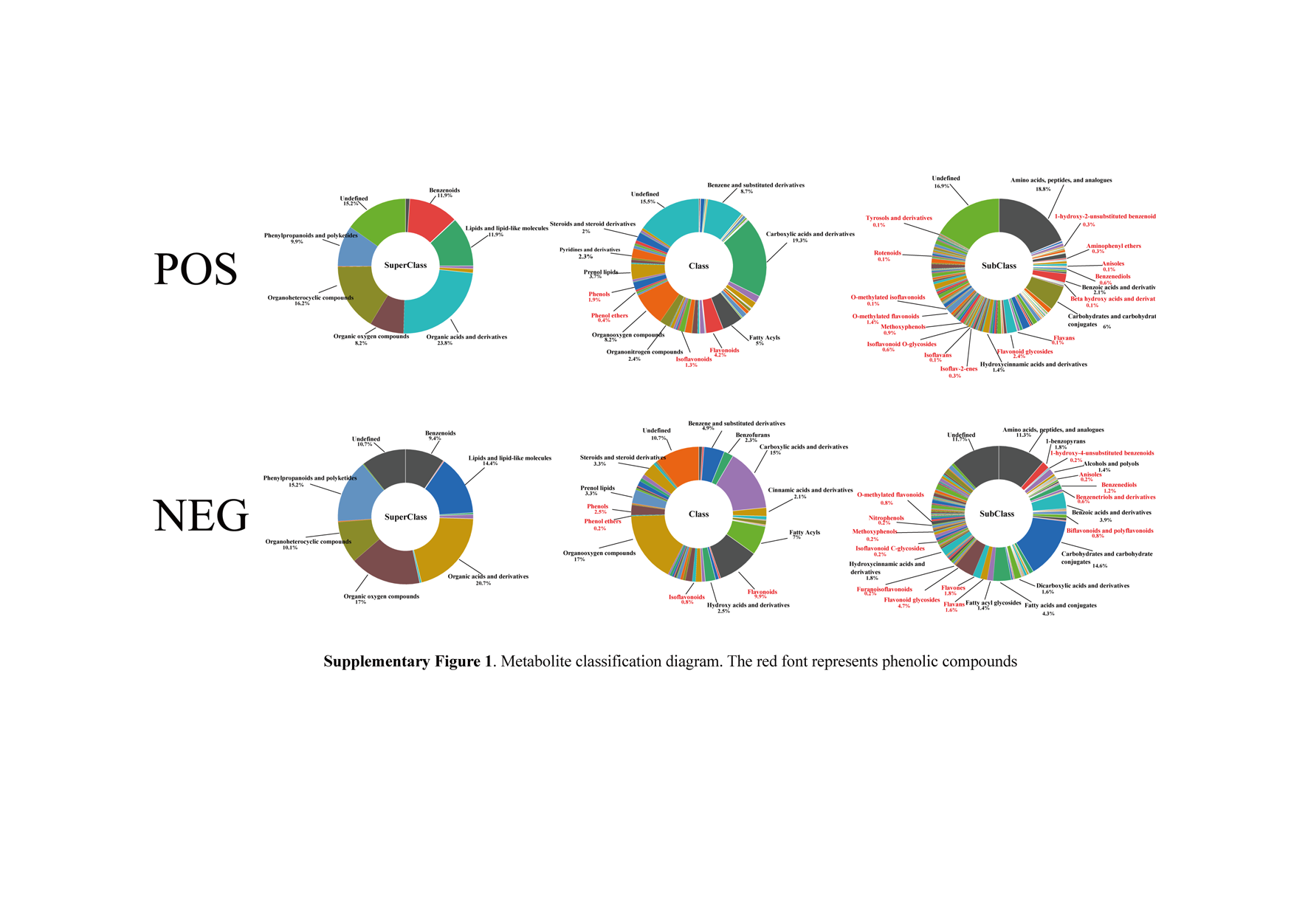

Supplement: Supplementary file 1 [file Image_1.TIF]
