# Supplementary material for: Fagopyrum dibotrys extract alleviates hepatic steatosis and insulin resistance, and alters autophagy and gut microbiota diversity in mouse models of high-fat diet-induced non-alcoholic fatty liver disease
Source: Front Nutr. 2022 Nov 14;9:993501. doi: 10.3389/fnut.2022.993501 (PMC9704541; doi:10.3389/fnut.2022.993501)
Supplement: Supplementary file 2 [file Table_1.docx]

| Composition  Calorie percentage (kcal%) | A normal diet | A high-fat diet | A normal feed mixed  FDE diet | A high-fat feed mixed FDE diet |
| --- | --- | --- | --- | --- |
| Protein | 23.07% | 19.8% | Normal feed ：FDE=9:1  (mass ratio) | High-fat feed ：FDE=9:1  (mass ratio) |
| Carbohydrates | 65.08% | 31.7% |  |  |
| Fat | 11.85% | 48.5% |  |  |

**Supplementary Table 1** The feed composition table of each diet.
