# Supplementary material for: Fagopyrum dibotrys extract alleviates hepatic steatosis and insulin resistance, and alters autophagy and gut microbiota diversity in mouse models of high-fat diet-induced non-alcoholic fatty liver disease
Source: Front Nutr. 2022 Nov 14;9:993501. doi: 10.3389/fnut.2022.993501 (PMC9704541; doi:10.3389/fnut.2022.993501)
Supplement: Supplementary file 3 [file Table_2.docx]

| **Gene Name** | **Forward** | **Reverse** |
| --- | --- | --- |
| *Gapdh* | CAGTGGCAAAGTGGATTGTTG | TCGCTCCTGGAAGATGGTGAT |
| *Ctsb* | CTCATGTAGGCTGCTTACCATA | TCTCCTTCACACTGTTAGACAC |
| *Atp6v0d1* | GCGGGCTGATGACTATGAACAGG | CGGTCCTCCAGGGTCTTGTCTC |
| *McoLn1* | TCCAGCTGAAGACAATTAACCT | GTGCGCTTTATTGTCAAATGTG |
| *Pparα* | AAGACTACCTGCTACCGAAATG | AACATTGGGCCGGTTAAGA |
| *Cpt1* | GAAGTGTCGGCAGACCTATTT | GTCCTCCTCTCTATATCCCTGTT |
| *Acc* | ACATTCCGAGCAAGGGATAAG | GGGATGGCAGTAAGGTCAAA |
| *Fasn* | AGACCCGAACTCCAAGTTATTC | GCAGCTCCTTGTATACTTCTCC |
| *Scd1* | CAACTTCACCACGTTCTTCATC | CCCGTCTCCAGTTCTCTTAATC |
| *Srebp1* | CATCGACTACATCCGCTTCTT | CACCAGGTCCTTCAGTGATTT |
| *Cd36* | GGAGTGCTGGATTAGTGGTTAG | GCTGTGAGCAGACGTATAGAAG |

**Supplementary Table 2** The primers used for qRT-PCR assay.
